# The clinical effect evaluation of multidisciplinary collaborative team combined with palliative care model in patients with terminal cancer: a randomised controlled study

**DOI:** 10.1186/s12904-023-01192-7

**Published:** 2023-06-13

**Authors:** Yu-Jing Liu, Li-Ping Wu, Hong Wang, Qing Han, Shu-Na Wang, Jing Zhang

**Affiliations:** 1 Department Of Geriatrics, Shijiazhuang People’s Hospital, Shijiazhuang, 050000 China; 2 Hebei University Of Chinese Medicine, Shijiazhuang, 050000 China

**Keywords:** Multidisciplinary collaborative team, Palliative care model, Quality of life

## Abstract

**Objective:**

To evaluate the clinical effect of a multidisciplinary collaboration team combined with a palliative care model in patients with terminal cancer.

**Method:**

A total of 84 patients diagnosed with terminal cancer in our hospital were included and randomly divided into an intervention group and a control group, with 42 cases in each group. Patients in the intervention group were treated by a multidisciplinary collaborative team combined with the palliative care model, and patients in the control group were treated by routine nursing intervention. The Self-rating Anxiety Scale (SAS) and the Self-rating Depression Scale (SDS) were used to evaluate negative emotions and anxiety and depression of patients before and after intervention. The Quality of Life Scale (European Organization for Research and Treatment of Cancer [EORTC] QLQ-C30) and Social Support Scale (SSRS) were used to evaluate the quality of life and social support of patients. This study has been registered in 13/01/2023 (ClinicalTrials.gov Identifier: NCT05683236).

**Result:**

The general data of the two groups were comparable. After intervention, the SAS (43.7 ± 7.4 vs. 54.2 ± 9.3) and SDS scores (38.4 ± 6.5 vs. 53.1 ± 8.4) of the intervention group were significantly lower than those of the control group. The total SSRS score, subjective support score, objective support score and utilisation of support of the intervention group were significantly higher than those of the control group (*P* < 0.05). The overall quality of life score of the intervention group was higher than that of the control group, and the difference was statistically significant (79.5 ± 4.5 vs. 73.2 ± 3.6, *P* < 0.05). The scores of each functional scale were significantly higher than those of the control group (*P* < 0.05).

**Conclusion:**

Compared with conventional nursing, the application of the multidisciplinary collaborative team combined with tranquilisation therapy in patients with terminal cancer can significantly reduce the anxiety and depression of patients, enable patients to obtain comprehensive social support, and effectively improve the quality of life of patients.

**Trial registration:**

ClinicalTrials.gov Identifier NCT05683236, 13/01/2023, Retrospectively registered.

**Supplementary Information:**

The online version contains supplementary material available at 10.1186/s12904-023-01192-7.

## Introduction

Cancer is one of the main causes of death in the world. With population growth and global ageing, cancer has become many countries’ main cause of premature death and reduced life expectancy. Related studies predict that in 2022, China will have about 4.82 million new cancer cases, and 3.21 million people will die from cancer [1]. Most patients with cancer were found to have entered advanced stages and become patients with end-stage cancer. The curative effects of traditional treatment, such as surgery, radiotherapy and chemotherapy, and interventional therapy for patients with end-stage cancer, are not good. It is toxic and comes with side effects and a considerable cost. It is reported that 80% of patients with advanced cancer in China have received this kind of “overtreatment” and have completed the last journey of life in pain [2]. This is not only not conducive to improving the quality of life (QOL) of patients but also directly leads to an increase in the mortality rate of patients with cancer and brings a heavy burden to families and society.

The psychological status of patients with terminal cancer is often complex. Due to the fear of cancer and the lack of correct understanding of the disease, patients have serious psychological pressure, which further aggravates the occurrence of negative emotions such as depression and anxiety and has a great impact on the QOL and body and mind [3]. Palliative care is not only for end of life, it is a humanised medical treatment service model that provides palliative and supportive care for patients in the end-stage and their families and provides psychological and physiological care for patients and their families [4]. Studies, domestic and abroad, have shown that palliative care has a positive effect on patients at the end of the disease. It helps alleviate patients’ physical pain, improves their mood and QOL before death, and ensures that patients walk through the final stage of life with dignity [5, 6]. Different from palliative treatment, palliative care gives life an “end with temperature”. With the progress of the concept of life and death in society, more and more patients and their families have accepted the concept of care.

The palliative care model is to establish a team through multidisciplinary cooperation to alleviate the physical and psychological pain of patients and improve their QOL. It is usually composed of medical staff, volunteers, physiotherapists, and psychological personnel. But there are still deficiencies in practice, such as imperfect team structure, insufficient communication between doctors and patients, and lack of professional knowledge and skills of medical staff, resulting in low quality of care [7]. Therefore, this study proposed a multidisciplinary collaborative team combined with a palliative care model and proposed to establish a team composed of medical staff in various disciplines to make up for the defects of a conventional nursing team. This study applied the multidisciplinary collaboration team combined with a palliative care model to patients with terminal cancer to evaluate its clinical effect.

## Subjects and methods

### Study subjects

**Fig. 1:**
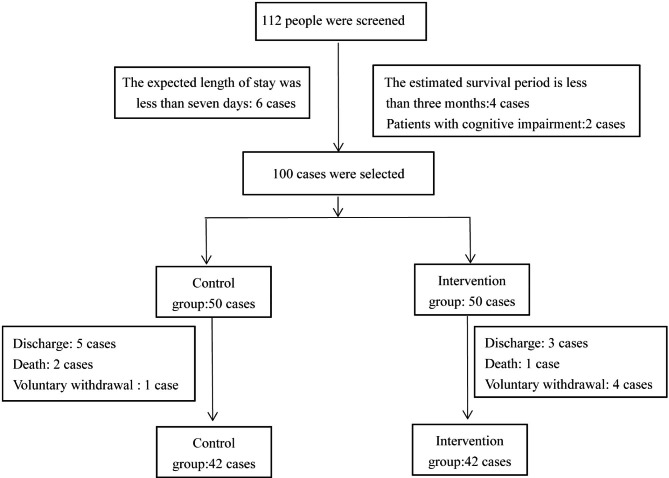
Participant flow chart

This study is a randomized controlled trial (parallel design). 100 patients with terminal cancer hospitalised in the oncology department of our hospital was selected to participate in this study. This study adheres to CONSORT guidelines. Participants were randomly assigned to different groups by a fixed researcher. The subjects were numbered according to the order of admission time and divided into a control group and an intervention group according to a random number table.First, number 100 patients from 1 to 100. Then look up the random number table, start from any row and any column in the random number table, read 3 digits as a random number, and correspond the random number with the number. Then all the selected random numbers are numbered from small to large (1-100). We specify that the control group is numbered 1 ~ 50, and the intervention group is numbered 51 ~ 100. Finally, 84 patients completed the study, with 42 cases in each group.The sample size calculation formula is as follows: n = Z ^2^ × (P × (1-P))/E^2^. Z was confidence interval, n was sample size, d was sampling error range, **σ** was standard deviation, generally be 0.5. E was the standard deviation of the sample mean multiplied by the value of z, that is, the total error.P was the proportion of the target population in the total population.After calculation, the sample size required for this study was 80 cases, 40 cases in each group. Two groups of patients were placed in different wards to avoid information contamination. Inclusion criteria: (1) Patients were diagnosed as terminal by imaging, pathology and clinical manifestations; (2) the expected length of stay was more than seven days; (3) patients with clear consciousness, good communication and understanding ability; (4) voluntary participation in this study and signing of informed consent; (5) the estimated survival period is more than three months. Exclusion criteria: (1) Patients with cognitive impairment and mental illness; (2) those who were seriously ill and could not cooperate with the completion; (3) patients with severe communication disorders. Participant flow is shown in Fig. 1.

### Study method

#### Control group intervention measures

The control group was given routine nursing intervention, strictly in accordance with a routine treatment process, monitoring the vital signs of patients, giving basic nursing, health education, diet guidance and so on. They were given appropriate psychological guidance, such as guiding patients to understand their condition further and guiding patients’ families to provide patients with reasonable psychological intervention. The support tried to vent the negative emotions of patients, maintain the maximum stability of the patient’s physical and mental state, and control the disease.

#### Intervention group intervention measures

The patients in the intervention group received multidisciplinary collaborative team care combined with palliative care as follows:

(1) Establish the multidisciplinary team. Team members were from multiple disciplines, mainly composed of clinicians, head nurses, and specialists in tumours, nutrition, rehabilitation, psychology and other areas. The whole team was led by case managers. Team division was as follows: The attending physician was mainly responsible for palliative treatment and symptom control of patients. The head nurse mainly managed the whole nursing team, supervised the implementation of the programme and was responsible for the nursing quality of the whole team. The case manager was mainly responsible for team coordination and arranging meetings to evaluate nursing work. The nurse team developed detailed programmes according to the arrangements of the head nurse, participated in meetings in a timely manner and guided clinical nursing work. Psychologists provided training on psychological counselling to nurses and supervised and guided nurses to carry out psychological support interventions once a week. The doctors and nurses participating in this study received training from nutrition experts, medical experts and psychologists to improve the implementation quality of the intervention measures.

(2) Evaluate the condition and make nursing plans. The medical experts evaluated the patients’ expected survival period and determined that the patients could enter the stage of palliative care. The case manager conducted a comprehensive assessment of the patients. After the assessments were completed, the results were sent to other team members. The other members of the team completed the specialist assessment within 24 h, and the first multidisciplinary consultation was conducted within 48 h to jointly develop the diagnosis and treatment plan. The medical experts provided guidance and consultation for the implementation of the nursing plan and inspected the ward once a week to find and correct the errors.

(3) Specific measures of the nursing programme.

① Health education: After teamwork to develop personalised education programmes, doctors and nurses carried out specific operations to strengthen patients’ cognition in a way and frequency that patients could accept. Doctors carried out health education for patients to avoid patients giving up their lives, encouraged them to actively cooperate with medical staff, and instructed patients on how to face the disease with a brave and strong attitude and actively fight the disease. Nurses conducted daily psychological counselling for patients, learnt their inner thoughts through communication with patients, and provided timely comfort and encouragement. An attention-shifting method can be used to alleviate the patient’s attention to the disease, effectively improving internal depression and avoiding depression. For patients with anxiety and loneliness, family members were instructed to accompany and care.

② Comfort care: For patients with terminal cancer in the ward for a long time, nurses should do their ward nursing work to ensure that patients are in a comfortable state. Reasonable control of indoor temperature and humidity is necessary to ensure that warm, light conditions permit properly dressed patients to feel warm. Nurses need to ensure that patients are kept clean and tidy in personal hygiene. They would regularly assist patients in turning over while giving patients sufficient respect.

③ Pain care: Physical pain will directly affect the patient’s physical and mental state. In order to alleviate the patient’s physical pain, it is necessary to give patients pain care. Clinicians should regularly evaluate the patient’s physical pain and give reasonable analgesic drugs to patients. Nurses should closely observe the medication response of patients. At the same time, it is necessary to observe and record patients’ physical pain daily and take effective pain control measures according to the actual situation to lay the foundation for improving patients’ comfort [2].

④ Dietary care: The clinical nutritionist formulated appropriate nutritional diets according to the nutritional status and personal preferences of patients, provided nutritional support for patients, and followed up with patients once a week after discharge to improve their nutritional status.

⑤ Psychological and social support care: Two nurses with psychological counselling qualifications in the team used the anxiety and depression scale to evaluate the psychological and emotional status of patients and referred to “The Questionnaire Survey on Awareness of Palliative Care” by the Department of Elderly Section of the union medical college hospital. The content mainly included the patient’s medical history and general situation, the needs and awareness of patients and their families for palliative care, the choice of patients and their families for the final treatment plan, and the needs of families for grief counselling. This was done to educate the patients and their families on the acceptance of death, coping ability, the assessment of patients on dying, and any future concerns. Timely professional counselling and intervention were undertaken to alleviate the patient’s adverse emotions. Family members were encouraged to involve themselves in the whole process, appreciate the good things and the most desirable people in the group, provide strong social support, comply with the wishes of the patients in the whole process, and strive to help the patients with their unfinished wishes so they could spend their final journey in peace. Patients were provided with goodbyes, thanks, apologies, and love opportunities to meet the emotional needs of patients and their families and comfort them.

⑥ Effect evaluation: The executors of the above care measures should do thorough periodic evaluations and feedback, and the case managers should pay attention to the problems in the care process and how to resolve them. The intervention of this study was from the patient’s admission to the hospital until the patient’s death. During this period, data collection (scale score) was conducted every other month, and the final data included in the analysis was the last score before the death of the patient. After the death of the patient, the family members were given psychological counselling for half a year.

### Observed indexes

The Self-rating Anxiety Scale (SAS) and Self-rating Depression Scale (SDS) were used to evaluate the patients’ adverse emotions, anxiety and depression before and after the intervention. The Quality of Life Scale (European Organization for Research and Treatment of Cancer [EORTC] QLQ-C30) and Social Support Rating Scale (SSRS) were used to evaluate the QOL and social support of patients.

#### Self-rating anxiety scale (SAS)

This involved 20 items, of which the 5th, 9th, 13th, 17th and 19th items were reverse scoring, and the 1–5 items were positive scoring. The items were scored according to Likert4 grade. The sum of the scores of each item is the total score of the scale. The scores were converted to a standard score: standard score = total anxiety score×1.25. The higher the score, the more severe the anxiety state. A score < 50 showed no anxiety, 50–59 showed mild anxiety, 60–69 showed moderate anxiety, and > 70 showed severe anxiety [8].

#### Self-rating depression scale (SDS)

There are a total of 20 entries, of which 10 are scored by forward and 10 by reverse. The entries are scored by Likert4 level. The sum of the scores of each item is the total score of the scale. The scores were converted to a standard score: standard score = total anxiety score×1.25. The higher the score, the more serious the depression. A score ≤ 50 showed no depression, 50–59 showed mild depression, 60–69 showed moderate depression, and > 70 showed severe depression [9].

#### Social support rating scale (SSRS)

The scale was compiled by Xiao et al [10]. with a total of 10 items, including three dimensions of subjective support, objective support and utilisation of support. The subjective support items are 1, 3, 4 and 5, the objective support items are 2, 6, and 7, and the utilisation degree of support items are 8, 9, and 10. Scoring method: 1–4 and 8–10 items; each item is a single choice, divided into 1–4 points. The 5th item is divided into A, B, C and D, four total scores. These are divided into 1–4 points for each item, from no to full support. The 6th and 7th items are divided into 0 points if “no source”, and those who answer “the following sources” have several sources. The total score of the scale is the sum of the scores of the three dimensions, and the higher the score, the greater the social support. Judgement criteria: A total score of ≤ 22 indicates a low level, 23–44 indicates a moderate level, and 45–66 indicates a high level, with a domestic norm score of 34.56 ± 3.73 .

#### Quality of life scale (EORTC QLQ-C30)

The Quality of Life Questionnaire-core30 (QLQ-C30), which was developed by the EORTC in 1993, was used to evaluate the QOL of patients with cancer. The Chinese version of the scale was translated and revised by Wan Chonghua. The Cronbach’s coefficient and test-retest reliability of each dimension of the scale were above 0.73, indicating that the scale has good reliability and validity and can be used for the QOL of patients with cancer in China [11]. QLQ-C30 consists of one overall QOL scale and five functional scales. The functional scales include physical function, role function, emotional function, cognitive function and social function. Rating criteria: After the scores of each part of the scale are converted to standardised scores, and the score range is 0–100. The higher the score on the scale, the better the overall QOL and functional status [12].

#### Statistical analysis method

SPSS 26.0 software was used for statistical analysis in this study. Quantitative data were expressed as mean ± standard deviation (‾×±s). Qualitative data were expressed as n (%). Single factor analysis of variance was used for comparison between groups. *P* < 0.05 indicated that the difference was statistically significant.

## Results

From the patient’s admission to the hospital until the patient’s death, data collection (scale score) was conducted every other month, and the final data included in the analysis was the last score before the death of the patient.The average time from the beginning of intervention to the death of 84 patients was 13 months (8–16 months). The trial ended after all participants died.

### Comparison of general data between the two groups of patients

There was no significant difference in gender, age, marital status, cancer type and other general data between the two groups (*P* > 0.05). This indicates comparability, as shown in Table 1.

**Table 1:** General information of two groups of patients

	Intervention group (*n* = 42)	Control group (*n* = 42)	*t*/*x*^2^	*P*
**gender**			0.207	0.649
Male (%)	26(61.9)	28(66.7)		
Female (%)	16(38.1)	14(33.3)		
**age**	65.4 ± 2.5	63.2 ± 1.2	5.431	0.345
marital status			1.251	0.535
Married (%)	38(90.5)	40(95.2)		
Unmarried (%)	1(2.4)	0(0.0)		
Widowed (%)	3(7.1)	2(4.8)		
**cancer type**			1.667	0.893
lung cancer (%)	18(42.9)	16(38.1)		
carcinoma of stomach (%)	10(23.8)	13(30.9)		
the other (%)	14(33.3)	13(31.0)		

Note: The mean between groups was compared by t test, and the percentage was compared by x^2^ test.*P* < 0.05 indicated that the difference was statistically significant

### Comparison of the two groups of patients with adverse emotions, anxiety and depression

Before the intervention, there was no significant difference in SAS scores and SDS scores between the two groups (*P* > 0.05). After the intervention, SAS (43.7 ± 7.4 vs. 54.2 ± 9.3) and SDS scores (38.4 ± 6.5 vs. 53.1 ± 8.4) in the intervention group were significantly lower than those in the control group. At the same time, compared with before intervention, the SAS (64.7 ± 10.2 vs. 43.7 ± 7.4) and SDS scores (67.3 ± 12.8 vs. 38.4 ± 6.5) in the intervention group after intervention were significantly decreased, while the SAS and SDS scores in the control group after the intervention were not significantly different from those before the intervention (*P* > 0.05). See Table 2 for details.

**Table 2:** Adverse emotions, anxiety and depression levels of patients in the two groups

Group	SAS Score			SDS Score	
	Pre-nursing	Post-nursing		Pre-nursing	Post-nursing
Intervention group(*n* = 42)	64.7 ± 10.2	43.7 ± 7.4*^#^		67.3 ± 12.8	38.4 ± 6.5*^#^
Control group (*n* = 42)	65.1 ± 12.3	54.2 ± 9.3		66.8 ± 10.5	53.1 ± 8.4

Note: Single factor analysis of variance was used for comparison between groups. P < 0.05 indicated that the difference was statistically significant.Compared with the control group (Post-nursing), * *P* < 0.05 ; compared with intervention group (Pre-nursing), # *P* < 0.05. SAS: Self-rating Anxiety Scale; SDS: Self-rating Depression Scale

### Comparison of social support between the two groups of patients

Before the intervention, there was no significant difference in the dimensions of social support between the two groups (*P* > 0.05), which indicates comparability. After intervention, the total score of SSRS, subjective support, objective support and utilisation of support in the intervention group were significantly higher than those in the control group (*P* < 0.05). Compared with before the intervention, the total score of SSRS and support scores of each dimension in the intervention group were significantly increased (*P* < 0.05), and the total score of SSRS in the control group was significantly increased (32.3 ± 1.2 vs. 25.0 ± 2.1, *P* < 0.05). This is shown in Table 3.

**Table 3:** Social support of the two groups of patients

Group	Subjective support score			Objective support score			utilization degree of support			SSRS total score		
	Pre-nursing	Post-nursing		Pre-nursing	Post-nursing		Pre-nursing	Post-nursing		Pre-nursing	Post-nursing	
Intervention group (*n* = 42)	12.5 ± 1.7	19.3 ± 1.2*^#^		8.1 ± 2.4	10.8 ± 2.0*^#^		6.5 ± 3.4	7.8 ± 3.1*^#^		26.3 ± 1.5	36.5 ± 0.7*^#^	
Control group (*n* = 42)	11.8 ± 2.4	16.4 ± 1.9		8.0 ± 2.3	9.6 ± 2.1		6.4 ± 3.0	7.2 ± 2.8		25.0 ± 2.1	32.3 ± 1.2^#^	

Note :Single factor analysis of variance was used for comparison between groups. P < 0.05 indicated that the difference was statistically significant. Compared with the control group (Post-nursing), * *P* < 0.05 ; compared with intervention group (Pre-nursing), # *P* < 0.05. SSRS: Social Support Scale

### Comparison of QOL scores between the two groups

Before the intervention, there was no significant difference in the QOL scale scores between the two groups (*P* > 0.05). After the intervention, the overall QOL scores of the intervention group were higher than that of the control group, and the difference was statistically significant (79.5 ± 4.5 vs. 73.2 ± 3.6, *P* < 0.05). The scores of each functional scale were significantly higher than those of the control group (*P* < 0.05). Compared with before the intervention, the overall QOL score and the scores of each functional scale in the intervention group were significantly increased (*P* < 0.05), and the physical function (62.3 ± 3.4 vs. 45.8 ± 5.6) and cognitive function (68.6 ± 3.8 vs. 51.3 ± 6.1) in the control group were significantly increased (*P* < 0.05). See Table 4.

**Table 4:** Quality of life of the two groups of patients

Group	physical function		role function		social function		emotional function		cognitive function		global quality of life	
	Pre-nursing	Post-nursing	Pre-nursing	Post-nursing	Pre-nursing	Post-nursing	Pre-nursing	Post-nursing	Pre-nursing	Post-nursing	Pre-nursing	Post-nursing
Intervention group (*n* = 42)	46.2 ± 5.3	65.4 ± 2.2*^#^	48.5 ± 6.4	60.5 ± 3.8*^#^	56.2 ± 4.3	75.4 ± 3.1*^#^	52.2 ± 6.2	67.4 ± 4.8*^#^	50.4 ± 5.7	73.7 ± 4.2*^#^	49.2 ± 3.2	79.5 ± 4.5*^#^
Control group(*n* = 42)	45.8 ± 5.6	62.3 ± 3.4^#^	48.2 ± 6.1	57.3 ± 4.1	52.6 ± 3.6	69.7 ± 5.3	53.5 ± 6.7	65.6 ± 5.2	51.3 ± 6.1	68.6 ± 3.8^#^	48.7 ± 4.5	73.2 ± 3.6

Note:Single factor analysis of variance was used for comparison between groups. P < 0.05 indicated that the difference was statistically significant. Compared with the control group (Post-nursing), * *P* < 0.05 ; compared with intervention group (Pre-nursing), # *P* < 0.05

## Discussion

With the improvement of medical technology in China, attention is now being paid to nursing work for patients with terminal cancer. Patients with end-stage cancer are generally in poor physical condition and often have severe pain accompanied by fatigue, nausea, vomiting and other symptoms, low QOL, and often require the assistance and care of others [13].

### Multidisciplinary team joint palliative care model

Palliative care can provide corresponding psychological help and psychological comfort to the family members of patients [14]. At present, there is still a big demand gap for clinical nursing care in China, so it is urgent for medical institutions and health workers to pay attention to it [15]. A multidisciplinary collaboration team is composed of professional medical personnel from different disciplines, which has the characteristics of clear division of labour and personalised diagnosis and treatment for characteristic diseases of patients who are terminal [16]. In addition, the multidisciplinary team combined with a palliative care model from the actual situation of patients, with patients and their families as the core, through the comprehensive analysis of their needs and demands, customised suitable, scientific and effective personalised nursing plan for patients [17].

### Impact on QOL

In this study, there was no significant difference in the scores of SAS and SDS between the two groups before intervention. After the intervention, the scores of SAS and SDS in the intervention group were significantly decreased and were significantly lower than those in the control group. It indicated that the multidisciplinary team cooperation combined with palliative care significantly alleviated the anxiety, depression and other negative emotions of patients with terminal cancer and reduced the degree of anxiety and depression. Moreover, the multidisciplinary team cooperation model had more advantages than the conventional nursing intervention.

Research shows that good social support can significantly affect the psychological behaviour of patients, such as enhanced treatment compliance and improved QOL [18]. In this study, we investigated the social support of patients with end-stage cancer and found that after the intervention of a multidisciplinary collaboration team combined with a palliative care model, the scores of social support in all dimensions of patients were significantly increased. The increase in the total score of social support was the most significant, and the scores of social support in all dimensions were significantly higher than those in the control group. The possible reason is that after receiving the intervention of the multidisciplinary team combined with a palliative care model, patients with terminal cancer have changed their past vague positioning or self-confidence in surrounding interpersonal relationships and their own social roles, thus alleviating the further deterioration of their psychological state. When patients with end-stage cancer suffer a series of attacks, their psychological reactions are easily affected by family members’ negative attitudes, family economic conditions and personal psychological quality. Therefore, helping them improve their subjective and objective support and support utilisation can improve the QOL of patients [19].

The results of this study showed that before the intervention, there was no significant difference in the QOL scale scores between the two groups. After the intervention, the QOL scale scores of the two groups were increased, and the overall QOL, physical, role, social, emotional and cognitive function scores of the intervention group were significantly higher than those of the control group. This shows that the application of a multidisciplinary collaborative team combined with a palliative care model in patients with terminal cancer can significantly improve their QOL and help alleviate the clinical symptoms of patients. In addition to physical pain, patients with end-stage cancer feel more fear, sadness and helplessness caused by approaching death, thus greatly reducing the QOL [20]. Palliative care can not only reduce the physical discomfort of patients but also provide psychological comfort and counselling in a timely manner, as well as provide appropriate death education and social and family support to meet the psychological and emotional needs of patients, thereby improving the QOL of patients.

### Prospects for palliative care

Palliative care can be further optimised and promoted from the following aspects, such as creating a family ward environment in the hospital and a good medical environment so that medical staff, hospice patients and family members live in a harmonious emotional space. This facilitates understanding and communication between doctors and patients. Palliative care also strengthens basic care and analgesic care to provide physical and psychological support. During hospitalisation, any minor changes in patients were closely observed, their pain was timely and correctly evaluated, and doctors received timely feedback.

### Advantages and limitations of this study

In this paper, the application of the multidisciplinary team combined with a palliative care model in patients with terminal cancer has achieved good clinical results. Compared with routine nursing, a multidisciplinary team combined with a palliative care model reduced the anxiety and depression of patients, enabled patients to obtain all-around social support, and effectively improved their QOL. This paper is limited to the lack of clinical samples, and it is difficult to obtain good extrapolation results. Moreover, the multidisciplinary collaborative team model needs more practice in the future to support its application effect. At present, the application research of this model is low. In addition, the blinding method was not used in this study because the nursing measures of the two groups of patients were very different, and the blinding method could not be realised.

## Electronic supplementary material

Below is the link to the electronic supplementary material.

Supplementary Material 1

## Data Availability

The datasets used and/or analysed during the current study are available from the corresponding author on reasonable request.
